# Evaluation of Prevalence of Hepatitis E Clinical Markers among Donors in Estonia

**DOI:** 10.3390/v15102118

**Published:** 2023-10-19

**Authors:** Tatiana Kuznetsova, Diana Moor, Gulara Khanirzayeva, Julia Geller

**Affiliations:** 1 Department of Infectious Disease Research, National Institute for Health Development, 11619 Tallinn, Estonia; 2 North Estonia Medical Centre’s Blood Center, North Estonia Medical Centre Foundation, 13419 Tallinn, Estonia; diana.moor@regionaalhaigla.ee (D.M.); gulara.khanirzayeva@regionaalhaigla.ee (G.K.)

**Keywords:** hepatitis E virus, seroprevalence, blood donor

## Abstract

Hepatitis E virus (HEV) is now considered the most common cause of acute hepatitis worldwide. There are no published data about the prevalence of antibodies to HEV and RNA in donor sera in Estonia, and this precludes planning measures for preventing HEV proliferation through blood transfusion services. Here, were report data from an analysis of 1002 sera on the prevalence of anti-HEV IgG and IgM and the viral RNA. The antibodies were found in 48 donor sera (4.8%); of these, 40 (4%) harbored anti-HEV IgG, 15 (1.5%) contained anti-HEV IgM, and 7 donors had anti-HEV antibodies of both classes simultaneously. HEV RNA was not detected in any blood serum. Statistical associations of infection risk factors (gender, age, travel in the last six months, contact with pigs and/or wild boars in the last six months, consumption of thermally unprocessed/raw pork or boar meat, raw/unfiltered tap water or water from natural sources, unpasteurized farm dairy products, and unwashed berries and/or vegetables) were assessed. None of the listed factors were found to be associated with a higher or lower risk of anti-HEV antibody presence. At the same time, an increasing share of anti-HEV IgG carriers with age was found. The absence of HEV RNA in the analyzed donor plasma samples proves that HEV acute infection prevalence in Estonia does not exceed the average level of European countries. There is no urgent necessity to enter a requirement for a total screening of blood plasma for HEV RNA prevalence in Estonia.

## 1. Introduction

In addition to the well-known and widespread hepatitis A, B, and C, hepatitis E (HEV) also occurs in Estonia [1,2]. The World Health Organization considers HEV the principal cause of acute hepatitis worldwide [3]. HEV is a small, non-enveloped, positive, single-stranded RNA virus. The virus has four major genotypes: HEV1 and HEV2 are found in humans only, whereas HEV3 and HEV4 genotypes cause disease both in humans and in some mammals, including pigs as the main reservoir. The disease is usually self-limiting, but immunocompromised people may develop persistent chronic disease and are at risk for more serious liver complications. In pregnancy, HEV infection increases the risk of preterm birth, stillbirth, and death of both mother and fetus [3]. In countries with tropical and subtropical climates, HEV1 and HEV2 infection occurs mainly through contaminated water, while in countries with cold and temperate climates, the virus is suggested to spread mostly by eating raw pork or contact with pigs carrying the virus [4]. Evidence of the long-term survival of HEV on fresh and frozen strawberries has been reported [5]. Our previous studies have shown a high prevalence of HEV in Estonian domestic pigs and wild boars, which are considered the main reservoir of HEV [6]. We have also evaluated antiviral antibodies and performed HEV genotyping in the Estonian adult population of different risk groups (patients with non-A, non-B, and non-C hepatitis symptoms; patients subjected to hemodialysis; healthcare workers; pig farm workers; hunters; veterinarians; patients with suspected HEV infection; people who inject drugs) [6,7,8]. Almost all observed risk groups showed an increase in the prevalence of anti-HEV antibodies with aging. Genetic analysis of the HEV sequences derived from domestic pigs’ and wild boars’ samples showed they belonged to the HEV3 genotype. HEV sequences obtained from hemodialysis patients and patients with suspected HEV infection belonged to HEV1 and HEV3 genotypes, respectively. Currently, much attention is paid to research on the risk of hepatitis E infection through blood, blood products, or other donated organs. The spread of the virus through blood transfusion has been confirmed in Japan, France, and England [4]. HEV screening among blood donors is currently considered the only effective means of preventing such cases. Out of 28 EU member states, HEV RNA donor screening has been introduced in 8 countries (Ireland, the United Kingdom, France, the Netherlands, Germany, Spain, Austria, and Luxembourg); 2 countries refused to introduce routine screening (Denmark and Sweden), and 6 countries have carried out preliminary studies of the donated blood but have not yet made a final decision (Italy, Poland, Portugal, Malta, Greece, Belgium) [9]. Among the EU countries, the highest prevalence of HEV RNA in donated blood was recorded in France; Germany, the UK, the Netherlands, Belgium, and Sweden have a lesser but substantial HEV prevalence (ranging from 1:844 up to 1:8000 [9,10]). Finland (1:5784) [11], Poland (1:2109) [12], and the states of south-eastern Europe [13] have lesser HEV prevalence than western European countries. In Russia, the anti-HEV IgG antibody detection rates in the general population increase significantly with age: 1.5% in children and adolescents under 20 years old, 4.8% in adults aged between 20 and 59 years old, and 16.7% in people aged 60 years and older [14]. HEV seroprevalence varies between regions, with the highest rate observed in the Belgorod region (16.4% compared with the national average of 4.6%), which has the country’s highest pig population.

There is no comprehensive explanation for why France is the most unsafe country for HEV infection in Europe. However, there is an opinion about HEV overdiagnosis in France due to the broad use of automated diagnosis stations for total blood plasma screening for HEV RNA unavailable in other countries [15]. Moreover, the appearance of the Beijing Wantai Biological serodiagnosis system (Sanbio, Beijing, China) based on mu capture raises anti-HEV antibody detection sensitivity twofold or more. Using Wantai and other more traditional screening systems (e.g., the test system by Mikrogen Diagnostik, Neuried, Germany) precludes correct data comparison about anti-HEV antibody prevalence in donor plasma in different counties and in different time periods [16].

The first published report about the overall seroprevalence of HEV-associated markers in the blood of different groups of the Estonian population appeared in 2012 in a local medical journal [1]. In 2012 in Estonia, the diagnostics of HEV had not yet been performed, and the occurrence of anti-HEV antibodies in the general population had never been studied. In this pioneering research, 1253 serum samples were subjected to a retrospective study. Among these, 763 samples were from patients with acute non-A, B, or C hepatitis: 176 from hemodialysis patients, 163 from health care professionals (HCW), and 151 from healthy people. The samples were collected between 1994 and 2006. All serum samples were tested for the presence of HEV IgG; positive sera were tested for HEV IgM antibodies using the corresponding ELISA and confirmed with immunoblotting. Out of all sera, 3.2% (40/1253) were positive for anti-HEV IgG. Anti-HEV IgM was found in 32.5% (13/40) of the anti-HEV IgG-positive samples. Overall, 27 sera were only positive for anti-HEV IgG. The highest HEV antibody occurrence, 6.0%, was noted in healthy subjects, followed by 5.1% in the group of hemodialysis patients. For both healthcare professionals and patients with acute non-A, B, or C hepatitis, the occurrence of antibodies was 2.4%. A comparison of the estimates by age revealed the highest occurrence rate (5.9%) in those aged 60 years and over.

A study [8] carried out in 2018 aimed to determine the prevalence and genotyping of HEV in different groups of the Estonian adult population. A total of 1426 human serum samples were tested (763 patients with clinically diagnosed non-A/B/C hepatitis collected in 1994–2000, 176 hemodialysis patients, 282 patients with clinically diagnosed non-A/B/C hepatitis collected in 2011–2017, and 205 people who injected drugs (PWID)). The presence of anti-HEV antibodies was assessed using an ELISA and confirmed with immunoblotting. All anti-HEV-positive sera were analyzed for RNA using a qPCR. The amplified ORF2 region was sequenced and used for phylogenetic analysis. An antibody assay revealed 49 samples from 1426 (3.4%) with acute (17) or past (32) HEV infection. HEV RNA was detected in 10 anti-HEV IgM-positive samples, including 9 samples from patients with suspected HEV infection and 1 hemodialysis patient. Anti-HEV IgG was found in 7.8% of patients with non-A/B/C hepatitis (2011–2017), in 4% of hemodialysis patients, in 2.4% of PWID, and in 1.96% of patients with non-A/B/C hepatitis (1994–2000). All groups demonstrated a trend of anti-HEV seroprevalence increasing with age. Phylogenetic analysis of nine HEV RNA sequences revealed that three sequences belonged to HEV genotype 1 and six to genotype 3 (one sequence belonged to subgenotype 3a, two to subgenotype 3e, and three to subgenotype 3f) [8].

Proximity to Sweden, on the one hand, and to Poland and Russia, on the other, does not allow for unambiguous forecasting of HEV prevalence in Estonia, whereas the necessity of making a decision about HEV control in donor plasma is a serious concern of the public health authorities in this country. While the majority of worldwide hepatitis E viral infections that occur in people are from contaminated water or food sources, there has also been a steadily rising number of reported cases of transfusion-transmitted HEV (TT-HEV) in blood donation recipients [4]. For most, HEV infection is acute, self-limiting, and asymptomatic. However, immunocompromised patients, especially transplant patients, are at much higher risk for developing chronic infections, which can progress to cirrhosis and liver failure, along with overall increased mortality. Because of the rising trend of HEV serological prevalence among the global population and the fact that TT-HEV infection can cause serious clinical consequences among patients most in need of blood donation, the need for screening for TT-HEV has been gaining prominence as an important public health concern for both developing and developed countries [4].

In 2017, EU/EEA countries were at different stages in their surveillance and testing capacity for HEV, and the European case definition was not commonly accepted [17]. For 2005–2015, at least 22 countries could report on cases infected with HEV, either through formal surveillance or existing systems of laboratory notifications. The World Health Organization (WHO)’s global health sector strategy on viral hepatitis asks countries to establish surveillance for viral hepatitis, particularly in blood donors [18]. Therefore, Estonian public health authorities must make a decision in this area.

## 2. Materials and Methods

### 2.1. Serum Samples

A total of 1002 serum samples were collected from healthy donors by the North Estonia Medical Centre’s Blood Center between 4 February 2022 and 21 March 2022. Participation in this study was voluntary, and samples were taken during routine blood donation. Specific exclusion criteria were not applied (all available sera were analyzed). The catchment area of the North Estonia Medical Centre’s Blood Center covers county Harjumaa, where the Estonian capital Tallinn is located, and the neighboring counties: Läänemaa, Raplamaa, Järvamaa, and Lääne-Virumaa.

There are 5 blood centers in Estonia—2 in Tallinn (in fact, there is 1 center with 2 blood donation points), and 3 in other cities—Tartu, Kohtla-Järve, and Pärnu. Everyone who wants to donate blood can come to any blood center; there are no specific limitations by area. There are requirements for health, weight, and age. Usually, a donor should be between 18 and 60 years old, but permanent donors up to 65 years old can donate if there are no health concerns. Donors appoint themselves donation time, but the frequency of procedures must not exceed once every 2 months for men and once every 3 months for women. Taken together, these data allow for the conclusion that our study does not cover the whole area of Estonia evenly.

The sera were provided within 1 day after blood donation; storage at the blood center and transportation with filled questionnaires to the National Institute for Health Development (NIHD) were carried out at +4 °C. If analyzed within the day of transfer, the sera were stored at +4 °C until used. For long-term storage, they were deposited at −20 °C.

### 2.2. Serological Assays

Sera were tested using ELISA for anti-HEV IgM and IgG antibodies using commercial kits (recomWell HEV IgM or IgG, Mikrogen Diagnostik, Neuried, Germany). Immunoblotting for anti-HEV IgG and IgM (recomLine HEV IgG/IgM, Mikrogen Diagnostik, Neuried, Germany) was used for confirmation. The samples were considered positive if both assays rendered positive results. All manipulation and interpretation of data were carried out according to the manufacturer’s instructions. Serum samples with the presence of anti-HEV IgM with or without IgG were classified as acute infection. The sera positive for anti-HEV IgG but not for IgM were annotated as past infection.

### 2.3. Detection of Hepatitis E Virus RNA

HEV RNA was extracted from 140 µL of serum using a QIAamp Viral RNA mini kit (QIAGEN, Hilden, Germany). Extracted RNA was stored at −20 °C until use. Real-time PCR was performed with One-Step Takyon^®^ Ultra Probe 4X MasterMix (Eurogentec, Seraing, Belgium), a forward primer (5′-GGTGGTTTCTGGGGTGAC-3), a reverse primer (5′-AGGGGTTGGTTGGATGAA-3′), and a probe (HEX-5′-TGATTCTCAGCCCTTCGC-3′-BHQ). The reaction was run in 10 µL mix per tube containing 5 µL of RNA, 2.5 µL of One-Step Takyon^®^ Ultra Probe 4X MasterMix, and the primers and probe at concentrations of 300 and 200 nM, respectively. The following cycling conditions were used: 50 °C for 10 min, 95 °C for 3 min, followed by two-step cycling 45 times at 95 °C for 15 s and 60 °C for 30 s. The 1st WHO International Standard for Hepatitis E Virus RNA Nucleic Acid Amplification Techniques (NAT)-Based Assays, code number 6329/10, was applied to evaluate real-time RT-PCR results.

Briefly, the lyophilized plasma standard containing HEV RNA was solved in 500 µL deionized water. The resulting master solution nominally contained 250,000 IU/mL. The master solution was used for preparing series of three dilutions with a 10-fold step. The resulted dilutions were used for RNA purification (100 µL aliquots of the dilution were used; the final RNA sample had total volume 50 µL). The resulting RNA samples had the nominal concentrations 500,000 IU/mL (master solution), 50,000 IU/mL (1st dilution), 5000 IU/mL (2nd dilution), and 500 IU/mL (3rd dilution). These samples were analyzed using RT-PCR and the following C_t_ values were obtained: master solution—28.7; 1st dilution—31.4; 2nd dilution—35.6; 3rd dilution—38.0. Taking into account our former experience, Ct = 38.8 was taken as zero. Extrapolating these data, the sensitivity of the method was determined as 62.5 IU/mL.

### 2.4. Statistical Analysis

Statistical analyses were performed using chi-square test (χ^2^). If the variable value was less than 5, the Yates-corrected chi-square was used. Differences were considered statistically significant when *p*-value was ≤0.05.

## 3. Results

### 3.1. Seroprevalence of Anti-HEV Antibodies and HEV RNA

Each of the 1002 blood donors involved in this study answered a questionnaire. Of these, some donors did not disclose information about the following variables: 5—age; 8—gender; 30—travel; 10—contact with pigs and/or wild boars; 7—undercooked pork; 5—unboiled/unfiltered water; 13—consumption of unpasteurized dairy products. Among the investigated donors, there were 466 women and 528 men aged 18–64 years; the average age was 38.8 ± 10.3 years. Anti-HEV antibodies were detected in 48 samples (4.8%), of which 40 (4%) samples were positive for anti-HEV IgG, 15 (1.5%) were positive for anti-HEV IgM, and antibodies of both classes were detected in 7 donors (0.7%). All anti-HEV Ig-positive samples while assessed using the recomWell kit were blot-positive too. None of the samples contained HEV RNA at a detectable level. In our opinion, these data should be perceived as conclusive since all chemicals and technical approaches to collecting samples, their storage, and analysis of performance have been tested previously [8].

As risk factors for the disease, the following were considered: gender, age, travel in the last six months, contact with pigs and/or wild boars in the last six months, consumption of thermally unprocessed/raw pork or boar meat, unboiled/unfiltered tap water or water from natural sources, unpasteurized farm dairy products, and unwashed berries and/or vegetables. There was no statistically significant correlation between the presence of antibodies to hepatitis E and risk factors (Table 1).

### 3.2. Age

**Figure 1:**
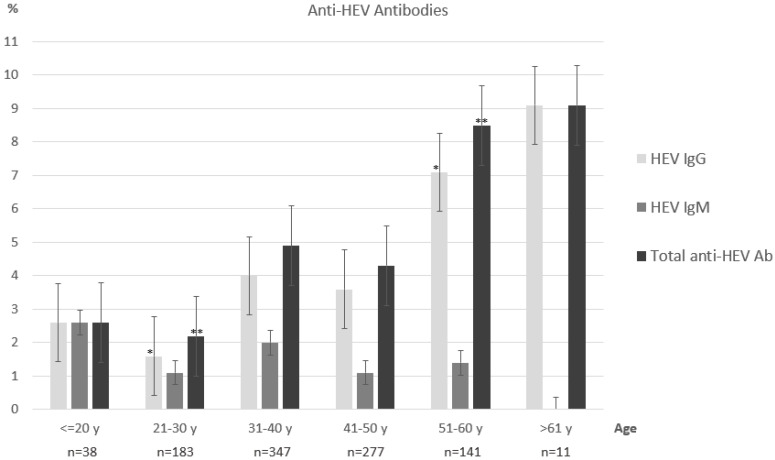
Anti-HEV antibodies in different age groups. Statistically significant differences are marked with asterisks (* *p* = 0.029; ** *p* = 0.019).

Based on the age reported by the donors, all participants were classified into six groups: “under 20 years old” (*n* = 38), “21–30 years old” (*n* = 183), “31–40 years old” (*n* = 347), “41–50 years old” (*n* = 277), “51–60 years old” (*n* = 141), and “over 60 years old” (*n* = 11). The prevalence of anti-HEV antibodies in different age groups is shown in Figure 1. Analysis of the results showed a trend towards an increase in the prevalence of anti-HEV IgG antibodies depending on age. Statistically significant differences were detected between age groups “21–30 years old” and “51–60 years old”: 3 IgG-positive sera out of 183 (1.6%) vs. 10 IgG-positive sera out of 141 (7.1%), *p* = 0.029; and 4 anti-HEV-positive sera (2.2%) vs. 12 anti-HEV-positive sera (8.5%), *p* = 0.019. The high proportion of anti-HEV Ig-positive samples in the age group over 60 may also be explained by the small number of samples in this group.

### 3.3. Gender

Anti-HEV antibodies were found in 4.5% of men’s and 4.9% of women’s serum samples (Table 1).

### 3.4. Traveling

Looking at the relationship between hepatitis E antibodies and travel to other countries, it was revealed that hepatitis E antibodies were present in 4.4% of travelers (Table 1). At the same time, hepatitis E antibodies were found in 5.3% of donors who had not left the country in the last six months. Among donors who visited foreign countries, anti-HEV IgM antibodies indicating acute infection were detected in individuals who had come back from Finland (two people), Cyprus (one), France (one), and Spain and Germany (one).

### 3.5. Contact with Animals

The next risk factor for hepatitis E infection is contact with domestic or wild pigs, as these animals are considered the main carriers of HEV. Anti-hepatitis E antibodies were detected in only 1 (4%) of 26 persons who had reported direct contact with animals in the last six months (Table 1). Among the individuals who did not report direct contact with pigs (*n* = 966), anti-HEV antibodies were found in 39 donors (4%).

### 3.6. Consumption of Unprocessed Pork

Looking at the relationship between the presence of hepatitis E antibodies and the consumption of thermally untreated/raw pork/wild boar meat, it was revealed that hepatitis E antibodies are present in 7.5% of consumers (Table 1). Hepatitis E antibodies were found in 4.8% of donors who did not report the consumption of undercooked or raw pork, according to the questionnaire answers.

### 3.7. Unboiled/Unfiltered Water

Anti-HEV antibodies were found in 4.8% of donors who consume unboiled/unfiltered tap water or water from natural sources (Table 1). However, hepatitis E antibodies were found in 5.3% of those who answered that they do not drink untreated/unfiltered water.

### 3.8. Unpasteurized Dairy Products

Looking at the relationship between the presence of hepatitis E antibodies and the consumption of unpasteurized farm dairy products, it was revealed that hepatitis E antibodies were present in 5.9% of consumers (Table 1). Of the donors who do not consume such food, hepatitis E antibodies were found in 7%.

### 3.9. Unwashed Berries and Vegetables

Anti-HEV antibodies were found in 7% of donors who noted the consumption of unwashed berries or vegetables (Table 1). Of the donors who answered that they always wash berries or vegetables before consumption, hepatitis E antibodies were found in 5.9%.

## 4. Discussion

Over the past few years, HEV infection has extended beyond endemic areas and occurs in most American and European countries. Although consuming contaminated water and food remains the main route of human HEV infection, blood transfusion is also shown to have a certain role in HEV morbidity in Europe [4,19]. There are no uniform regulations in the European Union defining the requirements for testing blood donors for HEV; therefore, the blood services of each country adopt their own regulations based on logistical, economic, and ethical issues.

The “gold standard” for molecular diagnosis of HEV infection is the direct determination of HEV RNA in blood, feces, and other fluids. HEV RNA can be detected and quantified using a nucleic acid assay (NAT). At the same time, there is no universally accepted standard solution to choose between individual donation (ID) NAT or minipool (MP) NAT protocols, so the practice varies widely among different laboratories worldwide. There are currently two approaches to HEV screening: total and targeted (selective) screening of all blood donors. Universal (total) screenings are in use in Ireland, the UK, and the Netherlands. A selective approach to screening implies stochastic examination of a certain share of the donated blood samples, taking into account individual risk of HEV infection. This approach is used in France, Austria, and Luxembourg. Although a selective screening strategy may seem more cost-effective, in practice, it requires more efficient management and imposes strict requirements on the feedback system [9]. Taking into account the high cost of any HEV survey system, our study aimed to provide an estimate of trends in the occurrence of HEV RNA and antiviral antibodies belonging to IgG and IgM classes in comparison to 2012 [1] and 2018 [8]. These data could be of interest in order to plan measures for HEV RNA screening of donated blood in Estonia.

First, we established the minimum required size of the examined group considering HEV prevalence in neighboring countries. In Latvia, there are no official or published data about HEV antibody and/or RNA prevalence in healthy donors; the only available data are 14 infected persons with clinical manifestation registered in 2014 and 8 persons in 2015. In 2021, in Lithuania, in a group of 100 donors, 3% produced positive blood samples containing IgG-class antibodies to HEV, and no anti-HEV IgM was found [20]. In 2006, in Sweden, in a group of 108 persons, 9.3% produced anti-HEV-positive donor blood samples containing IgG [21]. In 2016, in Sweden, this parameter reached 16% when measured in a group of 500 donors [22]. In 2023, in Finland, 7.4% of subjects were anti-HEV-positive donors with IgG-class antibodies, and 4.5% HEV-positive donors with IgM-class antibodies out of a group of 1009 donors [11].

The highest prevalence of HEV in Europe is registered in Corsica (France), where anti-HEV-positive individuals harboring IgG lass antibodies comprise 54% of the population [15]. A national report on the surveillance of HEV in France highlighted a sharp increase (9 to 2292) in the number of autochthonous cases reported to the public health authorities between 2002 and 2016. This apparent increase is likely due to (i) improved diagnostic tests and (ii) better awareness among physicians and in the general population, resulting in increased testing rather than a true epidemic situation. However, seroprevalence data (rates > 50%), the hospitalization rate per 100,000 inhabitants, and the total number of prescribed serological tests underline the hyperendemicity of HEV in southern France.

In Germany, in 2014, 6.8% of donor sera were found to be anti-HEV IgG-positive [23], whereas in 2012, the total share of anti-HEV IgG-positive individuals in the population comprised 16.8% [24]. In the Netherlands, in 2013, the total share of anti-HEV IgG-positive and IgM-positive donors found was 27% and 3.5%, respectively [25]. In Poland, there was a report of about 43–50% anti-HEV IgG individuals in 2018 [12,26]. On the other hand, the number of HEV RNA-positive samples was relatively low (1:2109) [12]. In Russia, there were 4.6% anti-HEV IgG-positive donors in 2023 [14].

Taken together, these communications allow us to hypothesize that the examination of a group of 1000 individuals can provide statistically relevant data about anti-HEV antibody prevalence in donors in Estonia.

On the other hand, the size of the examined group was highly likely to be insufficient for estimating HEV RNA. The highest prevalence in Europe of HEV RNA in donor blood samples (1:744) was registered in France in 2015 [9]. In Germany, in 2016–2017, this value was around 1:844 [9]. A complex analysis carried out in eight European countries in 2021–2018 reached a value of 1:3109 [9]. In Finland, in 2023, HEV RNA was found at 1:6000 [11], and in Sweden, in 2012, at 1:8000 [10]. Taking these figures into account, the absence of HEV RNA-positive samples in the examined group of donors looks quite probable and provides reliable evidence that acute HEV infection prevalence in Estonia does not exceed the average level of European countries.

The analysis of associations between the HEV infection risk factors—gender; age; travel in the last six months; contact with pigs and/or wild boars in the last six months; consumption of thermally unprocessed/raw pork or boar meat, unboiled/unfiltered tap water or water from natural sources, unpasteurized farm dairy products, and unwashed berries and/or vegetables—and anti-HEV prevalence produced no significant results. However, this is evidently explained by an insufficient number of HEV-positive donors. There was a slightly higher percentage of antibodies among people who had consumed unprocessed pork. We hypothesized that the Yates correction method may have produced overconservative results, and evaluation with an uncorrected chi-square test may produce another result. However, this hypothesis was not confirmed; *p* = 0.16 was found when significance was calculated this way. Three to five groups should be analyzed to make such a study informative, but this was not possible due to limited time and resources.

The choice of the serological test system for the screening of donor plasma has a critical impact on the specific figures of HEV-positive donors. Before 2016, test systems by Mikrogen Diagnostik (Neuried, Germany), Euroimmun (Lübeck, Germany), and DSI assays (Milan, Italy) were the most popular in Europe due to high sensitivity, although they were somewhat inferior in specificity to competitors [27]. A kit by Mikrogen Diagnostik was used in our previous studies [6,7,8]. This system was used in Lithuania in 2021 [20].

In 2016, a novel system by Wantai utilizing the mu capture principle became commercially available. There are many studies that have focused on the comparison of this novel system to the more traditional analogues. The Wantai assay resulted in a significantly higher seroprevalence for IgG and total Ig than the other assays [16]. The meta-analysis [27] reported a comparison of the Wantai system to analogues by Abbot, Adaltis, DiaPro, Mikrogen, MP, and others carried out in Austria, Belgium, Czech, Denmark, France, Germany, Italy, the Netherlands, Spain, Switzerland, and the UK. An average twofold higher seroprevalence was found in all countries when compared between data obtained with Wantai and other systems. In 2019, a meta-analysis [28] involving 125 original articles indicated that eight commercial serological systems for anti-HEV IgG and IgM screening are the most popular. In 56 studies, the Wantai assay was used (44.8%); in 18, DiaPro was used (14.4%); in 11, Abbott was used; in 11, Mikrogen was used (8.8% each); in 4, MP was used (3.2%); in 1, Adaltis was used; in 1, DSI was used (0.8% each); and in 64 studies, other/inhouse or undefined assays (51.2%) were used. A comparison of the system by Mikrogen diagnostics with Wantai was carried out in Spain [29]: the prevalence of IgG anti-HEV blood donors in Catalonia was 19.96% with Wantai and 10.72% Mikrogen.

The methods we used to detect HEV presence markers are not the most sensitive among the known ones. Higher sensitivity can be achieved by using kits for detecting viral antigen in the blood or by replacing blood with feces [3,4]. However, these methods were not available to us due to the high cost. In addition, when working with potential donors, fecal sampling is technically difficult and cannot be used routinely, which reduces the value of the data obtained in this way. To identify the role of HEV as an injective agent causing symptoms of liver failure, it would be advisable to analyze the parameters of ALT and AST aminotransferases in the blood of the studied patients, as recommended by the WHO [18]. However, these data were not provided to us by blood transfusion stations due to the lack of permission from the ethics committee for such a study.

Taken together, these reports indicate that the level of IgG and IgM anti-HEV seroprevalence in Estonia may be underestimated by about two times in comparison with data that could be obtained using the Wantai assay. However, even twofold greater seroprevalence than that found experimentally indicates Estonia as a country with a low level of anti-HEV antibodies in donors. Of note, IgG and IgM anti-HEV seroprevalence in Estonia did not change significantly in the periods from 2012 (3.4%) to 2018 (3.4%) and the present day (4.8%). This does not look like the picture of a 10-fold increase in HEV antibody seroprevalence in the period 2005–2015 in most EU counties described formerly [17]. Therefore, the Estonian authorities responsible for choosing the optimal approach to screening donors for HEV have a certain amount of time to make a decision. However, the gradual increase in the incidence of HEV in European Union countries may make introducing such a system crucial in the future.

## Figures and Tables

**Table 1:** Prevalence of anti-HEV antibodies in donors and estimate of putative risk factor significance.

Risk Factor	Anti-HEV IgG, %	Anti-HEV IgM, %	Total Anti-HEV Ab, %
Gender *			
Male (*n* = 528)	21/528 (4.0%)	8/528 (1.5%)	24/528 (4.5%)
Female (*n* = 466)	19/466 (4.1%)	6/466 (1.3%)	23/466 (4.9%)
Traveling *			
Yes (*n* = 387)	14/387 (3.6%)	5/387 (1.3%)	17/387 (4.4%)
No (*n* = 585)	26/585 (4.4%)	10/585 (1.7%)	31/585 (5.3%)
Contact with animals *			
Yes (*n* = 26)	1/26 (3.8%)	1/26 (3.8%)	1/26 (3.8%)
No (*n* = 966)	39/966 (4.0%)	13/966 (1.3%)	47/966 (5.1%)
Consumption of unprocessed pork *			
Yes (*n* = 107)	7/107 (6.5%)	3/107 (2.8%)	8/107 (7.5%)
No (*n* = 888)	33/888 (3.7%)	12/888 (1.4%)	41/888 (4.6%)
Consumption of unboiled/unfiltered water *			
Yes (*n* = 750)	29/750 (3.9%)	10/750 (1.3%)	36/750 (4.8%)
No (*n* = 247)	11/247 (4.5%)	5/247 (2.0%)	13/247 (5.3%)
Consumption of unpasteurized dairy products *			
Yes (*n* = 85)	5/85 (5.9%)	1/85 (1.2%)	5/85 (5.9%)
No (*n* = 904)	35/904 (3.9%)	13/904 (1.4%)	42/904 (7.0%)
Consumption of unwashed berries and vegetables *			
Yes (*n* = 755)	29/755 (3.9%)	14/755 (1.4%)	37/755 (7.0%)
No (*n* = 247)	11/247 (5.9%)	1/247 (1.2%)	11/247 (5.9%)

* No statistically significant correlation, *p* > 0.05.

## Data Availability

The data presented in this study are available in this article.
